# The ProPrems trial: investigating the effects of probiotics on late onset sepsis in very preterm infants

**DOI:** 10.1186/1471-2334-11-210

**Published:** 2011-08-04

**Authors:** Suzanne M Garland, Jacinta M Tobin, Marie Pirotta, Sepehr N Tabrizi, Gillian Opie, Susan Donath, Mimi LK Tang, Colin J Morley, Leah Hickey, Linh Ung, Susan E Jacobs

**Affiliations:** 1 Women's Centre for Infectious Diseases, Bio 21 Institute, 30 Flemington Road, Parkville, Victoria 3052, Australia; 2 University of Melbourne Department of Obstetrics and Gynaecology, The Royal Women's Hospital, 20 Flemington Road, Parkville, Victoria 3052, Australia; 3 Murdoch Children's Research Institute, Royal Children's Hospital, 50 Flemington Road, Parkville, Victoria 3052, Australia; 4 Department of Medical Education, University of Melbourne, Grattan Street, Carlton, Victoria 3053, Australia; 5 Department of General Practice, University of Melbourne, 200 Berkeley Street, Carlton, Victoria 3053, Australia; 6 Neonatal Services, Mercy Hospital for Women, 163 Studley Road, Heidelberg, Victoria 3084, Australia; 7 Clinical Epidemiological and Biostatistics Unit, Royal Children's Hospital, 50 Flemington Road, Parkville, Victoria 3052, Australia; 8 Department of Paediatrics, University of Melbourne, Parkville, Victoria 3052, Australia; 9 Neonatal Services, The Royal Women's Hospital, 20 Flemington Road, Parkville, Victoria 3052, Australia

## Abstract

**Background:**

Late onset sepsis is a frequent complication of prematurity associated with increased mortality and morbidity. The commensal bacteria of the gastrointestinal tract play a key role in the development of healthy immune responses. Healthy term infants acquire these commensal organisms rapidly after birth. However, colonisation in preterm infants is adversely affected by delivery mode, antibiotic treatment and the intensive care environment. Altered microbiota composition may lead to increased colonisation with pathogenic bacteria, poor immune development and susceptibility to sepsis in the preterm infant.

Probiotics are live microorganisms, which when administered in adequate amounts confer health benefits on the host. Amongst numerous bacteriocidal and nutritional roles, they may also favourably modulate host immune responses in local and remote tissues. Meta-analyses of probiotic supplementation in preterm infants report a reduction in mortality and necrotising enterocolitis. Studies with sepsis as an outcome have reported mixed results to date.

Allergic diseases are increasing in incidence in "westernised" countries. There is evidence that probiotics may reduce the incidence of these diseases by altering the intestinal microbiota to influence immune function.

**Methods/Design:**

This is a multi-centre, randomised, double blinded, placebo controlled trial investigating supplementing preterm infants born at < 32 weeks' gestation weighing < 1500 g, with a probiotic combination (*Bifidobacterium infantis, Streptococcus thermophilus *and *Bifidobacterium lactis*). A total of 1,100 subjects are being recruited in Australia and New Zealand. Infants commence the allocated intervention from soon after the start of feeds until discharge home or term corrected age. The primary outcome is the incidence of at least one episode of definite (blood culture positive) late onset sepsis before 40 weeks corrected age or discharge home. Secondary outcomes include: Necrotising enterocolitis, mortality, antibiotic usage, time to establish full enteral feeds, duration of hospital stay, growth measurements at 6 and 12 months' corrected age and evidence of atopic conditions at 12 months' corrected age.

**Discussion:**

Results from previous studies on the use of probiotics to prevent diseases in preterm infants are promising. However, a large clinical trial is required to address outstanding issues regarding safety and efficacy in this vulnerable population. This study will address these important issues.

**Trial registration:**

Australia and New Zealand Clinical Trials Register (ANZCTR): ACTRN012607000144415

The product "ABC Dophilus Probiotic Powder for Infants^®^", Solgar, USA has its 3 probiotics strains registered with the Deutsche Sammlung von Mikroorganismen und Zellkulturen (DSMZ - German Collection of Microorganisms and Cell Cultures) as BB-12 15954, B-02 96579, Th-4 15957.

## Background

### The intestinal microbial community and probiotics

Late onset sepsis (> 48 hours after birth) is a frequent complication in very preterm infants with high mortality and morbidity. In 2003, the Australian and New Zealand Neonatal Network (ANZNN) reported an incidence of 23% in very low birth weight infants (VLBW, less than 1500 grams) and 31% in extremely low birth weight infants (ELBW, less than1000 grams), with 18% mortality [1]. Preterm infants are more susceptible to infection as they have an immature immune system, which may in part be due to the abnormal development of their gastrointestinal microflora (microbiome). Molecular microbiological studies estimate that the mature human intestinal microbiome encompasses several hundred species of microorganisms with concentrations approximating 10^12 ^viable microbes per gram of faecal content [2]. This microbiome performs numerous important functions, including the digestion of macro and micronutrients, regulation of host fat storage, the metabolism of endogenous and exogenous compounds, immunoregulation and limiting colonisation with pathogenic microbes [3,4]. Disturbance of the gastrointestinal tract microbial environment may predispose individuals to a variety of diseases ranging from inflammatory bowel disease to allergy and obesity [4-7].

Colonisation of the aseptic intestine occurs rapidly after birth in healthy term infants. The intestinal microbial community is acquired from the birth canal and close parental contact after birth and is subsequently modified by diet [8]. Preterm infants acquire colonizing bacteria from the intensive care environment rather than their mother's vaginal canal, skin surface and milk [9]. Caesarean section delivery may delay infants' acquisition of diverse commensal flora by up to a year compared to vaginally delivered infants [10] and delivery by caesarean section occurs more frequently in preterm births (> 50% caesarean section rate for VLBW infants) [11]. These infants often receive antibiotics perinatally to prevent acute sepsis, and after birth for clinically suspected or definite culture positive sepsis, which further alters the composition of intestinal bacteria. Microbial diversity in the intestine of preterm infants, as assessed by culture-dependent methods, decreases with prolonged hospitalization [9,12-14]. Preterm infants also have delayed colonization with healthy commensals, such as *Lactobacillus *and *Bifidobacterium *species, which may lead to altered function of the gut microbial community, particularly the immune functions [9,15-19].

Probiotics are defined as live microorganisms, which when administered in adequate amounts may confer health benefits on people with specific illnesses [20]. Postulated mechanisms by which they may protect the host from gastrointestinal and urinary infections include: increasing resistance of the mucosal barrier to migration of bacteria and their toxins by strengthening intestinal cell junctions, modification of host response to microbial products, augmentation of immunoglobulin A mucosal responses, enhancement of enteral nutrition to inhibit the growth of pathogens; production of bacteriocins (small proteins which kill bacteria); and competitive exclusion of potential pathogens [21-25]. It has been hypothesized that very preterm infants, who have less microbial diversity in their GIT, may benefit from colonization with exogenously administered probiotics. Depending on the strain(s) administered, these probiotics could potentially induce a similar microbial community to that of the term infant or adult gastrointestinal microbiome [25]. The mechanisms by which probiotics may prevent the development of allergic disease are suggested to include all of the probiotic effects listed above as well as the induction of immune regulatory pathways

### Evidence from clinical trials of probiotics for prevention of sepsis and NEC in preterm infants

Several meta-analyses and systematic reviews of randomised controlled trials (RCTs) of probiotic use in preterm infants have been published in recent years [26-29]. The most promising results relate to supplementation with probiotics to prevent necrotising enterocolitis (NEC) and to reduce all-cause mortality.

Necrotising enterocolitis (NEC) is a relatively rare complication of prematurity. The incidence is inversely related to gestational age at birth. Overall, 88% of all reported cases in Australia in 2005 occurred in infants born before 32 weeks of completed gestation [1]. It is the most serious gastrointestinal problem of preterm infants and can rapidly progress from gastrointestinal dysfunction to bowel perforation and death in those most severely affected. Worldwide, the incidence is reported to be 1-3/1,000 live births and NEC affects approximately 10% of preterm infants born weighing less than1,500 grams [30]. Of those seriously affected, about half will require surgery to repair or excise diseased gastrointestinal tract and 25% will die. Long-term morbidity in survivors includes intestinal strictures, poor nutritional status and neurodevelopmental impairment [31-33]. No specific treatment exists for NEC; generally enteral feeds are ceased, with the administration of intravenous nutrition and broad spectrum antibiotics.

Cultivation and molecular-based studies have demonstrated quantitative and qualitative changes in faecal flora in the NEC prodrome; however, no single causative organism has been identified. Suggested pathogens have included bacteria (*E. coli, Clostridia perfringens, Enterobacter cloacae*, etc.), viruses (rota-, adeno-, corona-, noro-, etc.) and fungi (*Aspergillus *and *Mucor *spp.) [12,13,16,34-36]. NEC affected infants have also been shown to have lower species diversity in their gastrointestinal tract and this may limit the ability of their microbiome to perform its important immunoprotective functions.

The most recently published systematic review in 2,842 preterm infants concluded that enteral probiotic supplementation significantly reduces the incidence of severe NEC (Bell stage II or more) (typical RR 0.35, 95% CI 0.24 to 0.52) and mortality (typical RR 0.40, 95% CI 0.27 to 0.60) [29,37]. Other studies investigating probiotic use in very preterm infants have also reported favourable effects on the incidence of NEC, time to full enteral feeds, weight gain, colonisation rates and immune responses in the probiotic versus placebo group [38-43].

These studies support the hypothesis that augmentation of the intestinal microbiome of premature infants with exogenous probiotics (healthy organisms) may favourably alter the microflora resulting in an improvement in clinical outcomes. While these results are very promising, debate continues regarding the routine use of probiotics in preterm infants [44-46]. Many trials in these meta-analyses do not include the most vulnerable preterm infants, those born weighing less than 1,000 grams and before 28 weeks' gestation. In addition, there is a lack of data regarding the optimal strain(s), dose, time to start and duration of probiotic therapy, as well as the taxonomy and quality of currently available probiotics formulations and products. Further large clinical trials using products registered with microbial culture banks (for quality assurance) are required to resolve these issues and to support the development of probiotic products of pharmaceutical quality. Probiotic supplementation has not yet been evaluated in a population of Australian very preterm infants.

Results of studies investigating the use of probiotics to reduce the incidence or severity of sepsis have been equivocal. The Cochrane Database systematic review found no evidence of significant reduction of nosocomial sepsis (typical RR 0.90, 95% CI 0.76 to 1.07) [29], although some of the earlier individual RCTs demonstrated a difference in sepsis rates between probiotic and placebo groups [47,48]. The lack of effect reported in the Cochrane review may be due to the heterogeneity of the RCTs included in the review with regard to the variation in probiotic strains studied, the method of administration (dosage, frequency, duration, etc.) or differences in local nursery guidelines for the diagnosis and treatment of sepsis. Also, the sub-group of ELBW preterm infants who are most susceptible to late onset sepsis is under-represented as only small numbers of them were recruited to published trials.

These issues are being addressed by this large (1,100 participants), multi-centre, randomised, double-blinded, placebo controlled trial, which has adequate power to demonstrate clinically significant effects of the chosen probiotic mixture on the rate of late-onset sepsis in VLBW infants but not on NEC. It is also sufficiently powered to demonstrate any significant adverse effects in infants below 1000 g birth weight and less than 28 weeks' gestation.

### Evidence from clinical trials of probiotics for the prevention of allergic disease

An epidemic rise in allergic disease in "westernised" countries, especially food allergy and asthma, has occurred in parallel with many lifestyle changes [49]. Studies have linked alterations in the intestinal microflora in infants with the later development of allergic disease [50-52]. These abnormalities particularly involved *Bifidobacterium *species [53-57] and microbial diversity [12]. Probiotics are capable of inducing an intestinal microflora composition similar to that of a healthy breastfed term infant [58]. Therefore, they may have the potential to reduce allergic diseases by "normalising" the gastrointestinal tract microflora. Probiotics can also modulate immune function by altering dendritic cell [59], regulatory T cell [60,61], and T helper cell responses [62]. A Cochrane Library meta-analysis of probiotic supplementation for the prevention of allergic disease in term infants found a significant reduction in eczema in infants who received probiotics compared with those that did not [typical RR 0.82, 95% CI 0.70, 0.95] and further trials were recommended to determine the reproducibility of the results [63].

A more recent review and meta-analysis reported that a protective effect against allergic disease was only observed if probiotic supplementation were initiated in the prenatal period and continued postnatally, but not if probiotic supplementation were commenced postnatally [64]. However, there were only 4 small studies that administered probiotics postnatally, with 1 of these 4 studies reporting a beneficial effect from postnatal supplementation [65-68]. Therefore, further studies are required to determine if postnatal administration of probiotics might be beneficial for prevention of allergic disease.

## Aim of this study

The primary aim of this study is to determine the effect of probiotic organisms, ingested daily by VLBW preterm infants (below 1500 g and less than 32 weeks' gestation at birth) from shortly after birth, on the incidence of definite late onset sepsis, diagnosed after 48 hours of age and before term or discharge home, whichever occurs sooner. A secondary aim is to investigate the effects of probiotics on allergic diseases in this population.

## Methods/Design

### Trial Design

#### • Type of trial

This is a multi-centre, prospective, randomised, double blind, placebo controlled trial investigating the treatment of very preterm infants with a probiotic combination comprising *Bifidobacterium infantis, Streptococcus thermophilus *and *Bifidobacterium lactis *("ABC Dophilus Probiotic Powder for Infants^®^", Solgar, USA). The study powder contains 1 × 10^9 ^total organisms per 1.5 gm.

#### • Subjects and centres

A total of 1,100 subjects are being recruited and randomised from 12 perinatal centres around Australia and New Zealand (The Royal Women's Hospital, The Mercy Hospital for Women and Monash Medical Centre in Victoria; Royal North Shore Hospital, The Royal Prince Alfred Hospital, The Royal Hospital for Women, Liverpool Women's Hospital, Westmead Hospital and John Hunter Hospital in New South Wales; The Royal Hobart Hospital in Tasmania; and Auckland City Hospital and Christchurch Women's Hospital in New Zealand). Victorian recruits are offered the opportunity to participate in allergy assessments at 12 months' corrected age via maternal telephone questionnaire or assessment at The Royal Children's Hospital, Melbourne.

Parents are approached to consent to their baby's participation in the trial if they satisfy the inclusion and exclusion criteria (see below). Parents are provided with their own copy of the Patient Information and Consent Form and written consent is obtained from them. Infants enrolled from the Victorian centres are offered the opportunity to participate in allergy assessments at 12 months' corrected age, with consent for this being sought during the primary hospital admission.

#### • Duration of participation

Infants are given the probiotic or placebo from soon after the start of milk feeds until discharge home or term corrected age, whichever comes first. Victorian infants are followed until 12 months' corrected age.

#### • Timeline

Start of trial: October 2007

Recruitment period: estimated to be completed by March 2012

Time frame for statistical analysis: 6 months

Time frame for manuscript preparation: 6 months

The duration of the study is expected to be about 5 years from the beginning of subject recruitment, until the last data collection point.

#### • Study population

##### Inclusion criteria

a. Preterm infants both below 1500 g and less than 32 weeks' gestational age at birth

b. Enrolled within 72 hours of birth

##### Exclusion criteria

a. Major or suspected major congenital anomalies

b. Considered likely to die within 72 hours of birth

c. Parents from whom informed consent cannot be obtained. Babies of mothers who are already taking supplemental probiotics by capsule (not yoghurt, etc.)

### Outcomes

• **The primary outcome is the incidence of at least one episode of definite (blood culture positive) late onset sepsis before 40 weeks corrected age or discharge home, whichever occurs sooner.**

• The secondary outcomes are:

- incidence of clinically-suspected sepsis (blood culture negative sepsis)

- NEC, graded by modified Bell's criteria [37]

- mortality

- duration of the primary hospital admission

- number of courses of antibiotics

- number of days until full enteral feeds established (120 ml/kg/day or more for 3 consecutive days)

- In a subgroup of Victorian subjects at 12 months' corrected age:

(i) growth, including weight, length and head circumference

(ii) infections, hospital admissions and antibiotic courses

(iii) cumulative incidence of allergic disease, including atopic eczema, food allergy, and asthma/wheeze at 12 months as assessed by questionnaire [69,70]

(iv) immunological parameters including serum immunoglobulins, number of T- regulatory cells (Tregs) and levels of T- regulatory cytokines (Interleukin-10 and Transforming Growth Factor-β (TGFβ)) produced by mononuclear cells in-vitro [70].

(v) allergic sensitisation at 12 months - skin prick testing to a panel of common allergens (Egg white, milk, peanut, cat hair, grass pollen and house dust mite)

### Intervention and quality control

#### • Product description/Composition

The probiotic is the "ABC Dophilus Probiotic Powder for Infants^®^", Solgar, USA, containing 1 × 10^9 ^of total organisms/daily 1.5 g dose in a maltodextrin powder. This consists of *Bifidobacterium lactis, Streptococcus thermophilus *and *Bifidobacterium infantis*. The placebo looks identical to the probiotic and consists of maltodextrin. The maltodextrin powder contains 4 kcal/gram and is dairy free, gluten free and suitable for vegans. The daily dose is 1.5 g of study powder, irrespective of infant's weight or postnatal age.

#### • Confirmation of quality and safety of the probiotic

"ABC Dophilus Probiotic Powder for Infants^®^" is imported from Solgar, USA. This research team holds the import license obtained from the Australian Therapeutic Goods Administration Clinical Trial Notification (CTN) Scheme. The Solgar Company certify the quality of product through their established company protocols and ensure the correct transport of product to the pharmacy at The Royal Women's Hospital, Melbourne.

Taxonomy and quality of the probiotic organisms was confirmed by Dr. Barry Kiely of The Alimentary Pharmabiotic Institute, Cork, Ireland. Each batch of study and placebo powder is tested for the presence of pathogens using standard microbiological techniques and for the presence and quantitation of the probiotic organisms using real-time polymerase chain reaction techniques. This testing is undertaken at the microbiology laboratories of The Royal Children's and Women's Hospitals, Melbourne.

#### • Packaging and labeling with blinding

The probiotic and placebo powders are identically packaged by The Royal Women's Hospital Pharmacy Department, which holds the randomisation code. Each jar of study powder is identified with this unique code as well as the infant's medical record number following randomisation.

#### • Product handling

All study powder is supplied by Solgar^®^, USA. It is distributed by The Royal Women's Hospital Pharmacy Department to the individual study sites, where it is stored in a refrigerated dry environment at 4°C temperature. The investigators agree not to supply the study powder to any person except the infants participating in this trial.

### Management of subjects

#### • Route of administration

The study intervention/placebo is given orally or via gastric tube once daily, unless the feed volume is less than 3 mL when one level ProPrems trial measuring spoon of powder is administered in 1.5 mL feed twice daily. Freshly expressed mother's breast milk is the feed of choice followed by frozen breast milk, if fresh is not available. Banked breast milk is used when mother's milk is not available in centres with established protocols that use breast milk banking. Formula is used only when there is insufficient breast milk or if the mother chooses not to provide breast milk. Tube feeding is commenced as soon as possible in small and increasing amounts according to local protocols and the discretion of the treating neonatologist. Intravenous fluids and nutrition are used until approximately 120 ml/kg of milk is tolerated per 24 hour period. Feeding protocols aim to maximise the use of enteral feeding and minimise the use of central venous lines.

#### • Confirmation of probiotic colonisation

In Victoria, infants' stool carriage of probiotic species is documented to test for colonisation prior to the first dose of probiotic or placebo and then at 1, 4, and 8 weeks from treatment, and at 6 and 12 months' corrected age. Stool swabs are rotated in 200 μL of phosphate buffered saline (PBS) and DNA extracted as per the methods described by Furet, et al and Matsuki, et al. [71,72]. Extracted DNA is tested by quantitative real-time PCR for *Bifidobacterium infantis*, *Bifidobacterium lactis *and *Streptococcus thermophilus *using primers and probes directed at a 16S rRNA and 16S-23S integrated spacer gene respectively [73,74]. These methods are more sensitive than conventional culture techniques with detection limits of approximately 1 colony-forming unit/ml. This testing is undertaken at the Women's Centre for Infectious Diseases Molecular Microbiology Laboratory, The Royal Women's Hospital, Melbourne.

#### • Compliance

The probiotic study powder is administered, as ordered on the medication chart, until the baby reaches term corrected age or is discharged home from hospital, whichever comes first. Administration is monitored by the research team at each study centre.

#### • Allergy assessment at 12 months' corrected age

Infants enrolled at the 3 Victorian sites are eligible for the 12 month corrected age allergy assessment. This is coordinated by a study nurse and involves either a maternal telephone questionnaire or an assessment at The Royal Children's Hospital (RCH), Melbourne. Parents who consent to their infants' being assessed at 12 months' corrected age are asked to keep a written record of any infections, hospital admissions or allergic symptoms experienced by their child during this time period.

There are several components to the allergy assessment:

(i) A parental questionnaire is used to assess the incidence and severity of atopic eczema, food allergies and wheeze from term until 12 months' corrected age. This is a validated questionnaire which has been adapted from the Probiotic Eczema Prevention Study (Principal Investigator M. Tang) [70].

(ii) Where permission is given on the day, a blood sample (1 - 2 ml) is collected following the application of a topical anaesthetic (Emla 5%) to minimize distress to the child. Serum and peripheral blood mononuclear cells are isolated for future batched analysis of serum immunoglobulins, number of Treg cells, and in-vitro production of IL-10 and TGFβ levels. These studies will be performed at the Murdoch Children's Research Institute (M Tang, Allergy and Immune Disorders Research Group).

(iii) Skin-prick testing (SPT) is offered to determine sensitization to common allergens (Egg white, milk, peanut, cat hair, pollen, house dust mite (DerP1), 10 mg Histamine and a negative control). Participants with SPT responses ≥3 mm above the negative control are referred to the Paediatric Allergy Service for further assessment and management, including confirmatory oral food challenge if required.

#### • Study withdrawal

In the case of early withdrawal from the study, a research nurse records any reason given for withdrawal and attempts to collect as much data as possible on the Case Report Form. If Victorian parents refuse to continue the study treatment, they are asked if they are willing to participate in the 12 month follow-up questionnaires. If parents do not agree, they are not contacted any more. If participant withdrawal amounts to > 1% ie.11 subjects, 1% additional subjects will be recruited to compensate for missing data. Minimal data is collected on infants whose parents decline to be involved in the study, to ensure that participants are representative of the eligible population to ensure that the denominator can be estimated.

### Conduct of the trial

#### • Randomisation

This is achieved using variable block design constructed by the Clinical Epidemiology and Biostatistics Unit (CEBU) at the Murdoch Children's Research Institute, who are independent of the study investigators. Each block contains equal numbers of placebo and active allocation. Randomisation is stratified by unit. Each of the participating neonatal units is able to access the next sequentially numbered probiotic or placebo immediately after randomisation. Records are kept and audited to check that sequential randomisation is used to assign infants to either study group. All doctors, nurses, research assistants, laboratory staff and parents are blind to the randomised allocation. The key is held by the Chief Pharmacist at The Royal Women's Hospital and the trial statistician, both of whom are independent of the investigators and not involved in outcome assessment.

#### • Breaking of the code

All attempts to avoid breaking the code will be made for the duration of the study. The code break may be used by one of the 5 Chief Investigators (CI) directly in true emergencies after consultation with at least one other CI. In such cases the rationale must be documented, with immediate notification of the Project Leader. The code break will also be reported in the participant specific case report form (CRF) and events leading to the emergency breaking will be recorded in the serious adverse event (SAE) report form. SAEs will also be notified to all ethics committees and the Therapeutic Goods Administration [75].

#### • Data management

Hard copies of the study data are stored in locked filling cabinets at each study centre. Electronic data will be stored in the Department of Newborn Research at the Royal Women's Hospital, Melbourne on secure password protected computers for 25 years. Every effort will be made to obtain the highest volume of follow-up data.

### Statistical Aspects and Consideration

#### • Sample size calculation

Statistical advice has been provided on all aspects of study design, sample size and analyses by CEBU, who will assist in analysis at trial completion.

To reduce the incidence of culture proven sepsis from 23% to 16% (a 33% reduction) with a power of 0.8, α of 0.05, and a 2-tailed test, the estimated sample size is 530 per group. The trial will therefore recruit 1,100 infants over 5 years, based on our experience that 50% of parents consent to participation in this preterm population.

#### • Statistical Analysis

Intention to treat analysis: Data from all randomised participants will be considered in the intention-to-treat model (for the primary outcome).

Per protocol analysis: Per protocol evaluation will exclude data from participants who did not receive the intervention.

Interim analyses: An independent data monitoring committee (DMC) consisting of an independent paediatrician with expertise in neonatal sepsis, an epidemiologist, and two neonatologists with clinical trials experience has been appointed. Full interim analyses of all outcomes are being undertaken as data is received when 100, 400 and 700 recruits have completed the study. An additional interim analysis of mortality occurred when 200 participants reached term corrected age. Any adverse events, which on further assessment appear likely to be due to the probiotics, are reported to the clinical trial steering committee, the local hospital research committees at participating centres and where appropriate, the Therapeutic Goods Administration.

### Legal and Ethical Prerequisites

#### • Legal requirements

This study is carried out according to the *National Statement on Ethical Conduct in Research Involving Humans (March 2007) *produced by the National Health and Medical Research Council of Australia. This information has been developed to protect the interests of people who participate in research studies.

#### • Ethical aspects

##### Protection of the subject's confidentiality

Confidentiality of all study participants is maintained; codes for subject identification are utilised. Identifying or confidential data is kept in locked cabinets or password protected computer databases, accessible only by named investigators.

##### Informed consent

When the investigator has determined that the potential participant is eligible for the study, the study is described and explained verbally to his/her parent/legal representative. The investigator fully answers all questions. A copy of the information sheet is given to his/her parent/legal representative.

Written, informed consent is obtained from each participant's parent/legal representative by the investigator prior to enrolment in the study. The consent form is signed and dated by the parent or the infant's legal representative and the investigator. The consent form is completed in two copies: the first copy is given to the parents or guardians and the second is stored in the participants' medical record. No participant receives treatment before completion of the written informed consent.

##### Ethics Committee Approval

The study protocol has been approved by the institutional Human Research and Ethics Committee (HREC) of each participating centre. The initial approval was granted by The Royal Women's Hospital HREC. The RWH project approval number is 06/31. The local ethics committee is notified of all subsequent additions or changes in the study protocol. Regular notification of the ethics committees is also required for any serious adverse events during the clinical trial.

##### Declaration of Helsinki

This trial is being conducted according to the principles and rules laid down in the Declaration of Helsinki and its subsequent amendments.

#### • Termination of study

Should it prove necessary to discontinue the study permanently prior to completion, the chief investigators: Prof Suzanne Garland, Dr Tobin, Dr Jacobs, Dr Pirotta, and A/Professor Tabrizi will reach a consensus decision that this is required and sign a joint document to notify the investigators and additional contacts, including the HRECs, of the rationale. All study relevant documents will then be returned to The Royal Women's Hospital, VIC, and the study product will be destroyed or returned.

## Discussion

Late onset sepsis is associated with high mortality and morbidity in preterm infants. Abnormalities in their intestinal microbiota development, which has important immune functions, may explain their increased susceptibility to infection. Disturbance of the gastrointestinal tract microbial environment predisposes adults and children to disease, including inflammatory bowel disease, allergy and obesity [4-7]. It has been hypothesized that very preterm infants, who have less microbial diversity in their GIT, may benefit from colonisation with exogenously administered probiotics.

Probiotics are defined as live microorganisms, which when administered in adequate amounts may confer health benefits on the host [20]. The most recently published systematic review of probiotic supplementation in preterm infants concluded that enteral probiotics significantly reduces the incidence of severe Necrotising Enterocolitis and mortality [29,37]. Other favourable effects reported include reduced time to full enteral feeds, improved weight gain, colonisation rates and immune responses in the probiotic versus placebo group [38-43].

The effect of probiotics on the incidence or severity of sepsis has been equivocal. This may be due to the heterogeneity of the RCTs included in the most recent Cochrane review with regard to the variation in probiotic strains studied, the method of administration (dosage, frequency, duration, etc.) or differences in local nursery guidelines for the diagnosis and treatment of sepsis. Also, the sub-group of ELBW preterm infants who are most susceptible to late onset sepsis is under-represented in the review.

Whilst the above findings appear promising, debate continues regarding the routine use of probiotics in this vulnerable population [44-46]. Further large clinical trials using products registered with microbial culture banks (for quality assurance) are required to support the development of probiotic products of pharmaceutical quality. This large multi-centre, randomised, double blinded, placebo controlled trial has adequate power to demonstrate clinically significant effects of the chosen probiotic mixture on the rate of late-onset sepsis in VLBW infants. It is also sufficiently powered to demonstrate any significant adverse effects in infants below 1000 g birth weight and less than 28 weeks' gestation. More than 900 infants have been enrolled to date, therefore, we are on course to reach our target recruitment number of 1,100 participants and anticipate that results will be available in early 2012.

## Competing interests

**SMG, JMT, ST, MP, CJM, GO, SD, LH, LU, SEJ - **none to declare, **MLKT **is a member of medical advisory board Nestle Nutrition Institute; has given speaker presentations for various pharmaceutical companies, including Merck Sharp Dome and Alphapharm; and is co-investigator on a clinical trial funded by Danone.

## Authors' contributions

All authors assisted with the preparation of this manuscript. **SMG **conceived of the study and participated in its design. She also coordinates the trial, assists with acquisition of data and supervises the microbiological analysis. **JMT **was involved in study design, wrote the NHMRC Grant with SMG, CJM and MP, and the Victorian ethics applications, managed study powder logistics, drafted the original trial operation protocol with LU and the rest of the authors, and assists with trial co-ordination.

**MP **was involved in study conception, development of the protocol and is involved with the overseeing of the study. **ST **designed the assays used for the molecular microbiology testing of faecal samples and coordinates the ongoing testing of these samples. **GO **developed the intensive care operational protocol with SEJ, is a site investigator and involved with study recruitment. **SD **is the trial statistician.

**MLKT **contributed to study design in relation to allergic diseases and immunological outcomes (questionnaire, blood testing, SPT) and coordinates the clinical outcomes assessment and laboratory assays performed at RCH and MCRI. **CJM **was involved in the trial conception and design. **LH **drafted this manuscript for publication and was involved with study recruitment. **LU **assisted with drafting of the original trial operation protocol. She currently coordinates the trial with SMG and is responsible for data management. **SEJ **was involved in the study design and developed the intensive care operational protocol, is a site investigator with involvement in study recruitment, provides the neonatal clinical expertise for participating sites and assists SMG with the trial coordination. All authors have read and approved the final manuscript.

## Author's information

**SMG **MBBS MD FRCPA FRANZCOG *Ad Eundem *FAChSHM. Director of Microbiology and Infectious diseases, RWH. Expertise in clinical trials, evaluation of probiotics as interventions for various disease entities, as well as an interest in reducing neonatal sepsis.

**JMT **MBBS FRACP PhD GradCertUniTeach, Paediatric Nutrition and Gastroenterology. Senior Lecturer in Medical Education. Expertise in clinical trials and an interest in probiotics from infancy to adulthood.

**MP **MBBS is an academic general practitioner researcher with experience in probiotic research.

**ST **PhD is senior scientist at The Women's Centre for Infectious Diseases, RWH.

**GO **MBBS IBCLC FRACP. Head of Unit, Mercy Health Breast milk bank. Neonatal paediatrician with expertise in clinical trials and nutrition in preterm infants.

**SD **is Associate Professor at The Clinical Epidemiology and Biostatistics Unit, RCH.

**MLKT **MBBS, PhD, FRACP, FRCPA, FAAAAI. Allergist Immunologist, Director of Department of Allergy and Immunology, Royal Children's Hospital. Expertise in clinical trials, evaluation of probiotic effects on microbiota and immune function.

**CJM **MD FRCPCH Retired Professor of Neonatology

**LH **MB BCh BAO (Hons) MRCPI. Paediatrician with subspecialty training in Neonatology. Currently undertaking a Doctorate of Medicine Degree (MD) through research at Professor Garlands' molecular microbiology laboratory.

**LU **BSc(Hons). Project manager with expertise in multi-centre clinical trials.

**SEJ **MBBS, FRACP, MD. Neonatal Paediatrician and Director, Neonatal Nurseries. Expertise in clinical trials and an interest in neonatal outcomes, including sepsis.

## Pre-publication history

The pre-publication history for this paper can be accessed here:

http://www.biomedcentral.com/1471-2334/11/210/prepub
